# The Notification of Measles

**Published:** 1903-01-24

**Authors:** 


### THE NOTIFICATION OF MEASLES.

THE fact that measles had been the direct cause of the loss of 1,930 lives in a single year in London was ample excuse for the action taken by the County Council on Tuesday last in applying to this disorder certain sections of the Public Health (London) Act, 1891, referring to "dangerous infectious diseases." The Council hesitated to recommend that measles should be made a notifiable disease, being deterred no doubt by the heavy expense which such a course would have involved. The heavy mortality referred to above, connoting as it does the existence of a very large number of cases, suggests, what is indeed the truth, that the compulsory notification of so common a disorder as measles would turn out to be a far bigger affair than some people might on first thoughts imagine. How big we can only as yet estimate by comparison. In the absence of notification it is always difficult to ascertain the fatality of a disease. The number of deaths is, of course, known; but as to the proportion which exists between them and the sum total of the cases we know but little. A certain amount of information on the subject has, however, been collected from the returns made by the teachers of the public elementary schools and from other sources, and by comparing this with the number of deaths it has been made out that in London there are at least 13 cases for every death, ranging from 10 in the Strand to 21 per death in Paddington. All this, however, is an obvious under-estimation of the frequency of measles, for when we turn to Edinburgh, where for a considerable number of years the notification of this disease has been compulsory, we find that on an average there have been 32 cases to each death. If, then, we provisionally accept this proportion, it would appear that the numbers to be dealt with in London cannot fall far short of the enormous total of 61,760 cases of measles. For the sake of comparison it may be stated that the total number of cases of scarlet fever notified in London during the year 1900 (the year already referred to in regard to measles) was only 13,892. Moreover, in discussing the question of notification we must not forget what notification might lead to. In the minds of many it seems so obvious as hardly to need argument that if notification is to serve any good purpose its natural and reasonable outcome is the provision of hospital isolation for the cases notified, and we may safely presume that if notification were enforced this would soon be succeeded by a demand for the treatment at the public expense of the cases notified. Hence, the resolution presented to the London County Council on Tuesday is likely to be regarded by some as the thin end of a very large wedge, especially as whatever course may be adopted in regard to measles ought, in all reason, to be applied to the diminution of the useless loss of life which is constantly taking place from whooping cough.

It must be borne in mind, however, that, plain and obvious as it may seem to some that notification should lead to isolation, this is a sequence of events which by no means of necessity follows. Doubtless we shall be asked, What is the use of merely calling measles a "dangerous infectious disease," or of going to all the trouble and expense of notification, if we are to be content with the mere knowledge that the cases exist? But it would be a very narrow view of the functions of a public health department which should limit its dealings with epidemics to such an elementary proceeding as the attempt to protect the healthy by bottling up the infected. Every year and every day is bringing into greater prominence the fact that not only the activity of an infection, but the malignity of the disease produced by it, depends much upon the surroundings amid which the sufferer finds himself, and it is being borne in upon us that the true work of a sanitary authority is to maintain the conditions of health rather than to directly combat disease. In fact, the notification of disease becomes a matter of interest to the sanitarian, rather as a means of showing him where his measures have failed, than as merely indicating where to send the ambulance. When then we are asked, What good can the inclusion of measles in the list of "dangerous infectious diseases" do, seeing that we cannot possibly afford to isolate the cases in hospitals? we may fairly point out that in dealing with such diseases there are clauses in the Act which prohibit the exposure of infected persons and things; which enable the authority to enforce the cleansing and disinfection of infected articles and premises, and to offer compensation and give shelter during the process; which prohibit persons from knowingly letting houses in which infected persons have been lodging until such premises have been disinfected; and give many other powers by which the spread of infection may be checked. Notwithstanding then that isolation, which so many people have regarded as the sole aim and end of notification, is quite out of the question in regard to measles, the inclusion of this malady in the list of "dangerous infectious diseases" will place in the hands of the sanitary authorities many very effective means for its control.

